# Toward a national laboratory system for public health.

**DOI:** 10.3201/eid0707.017710

**Published:** 2001

**Authors:** M R Skeels

**Affiliations:** Oregon State Public Health Laboratory, Portland, Oregon 97207, USA. Michael.r.skeels@state.or.us


### Toward a National Laboratory System for Public Health

**Michael R. Skeels**
Oregon State Public Health Laboratory, Portland, Oregon, USA

Currently, there is no cohesive national system of laboratories to support community health activities. One vision for such a system would be a cooperative arrangement of public health, hospital and independent laboratories that address community needs (R.A. Martin, pers. comm., 2000; 1-3). The rationale for this system includes the following:

- Laboratory services for infectious and toxic agents are vital to community health.
- Information technology has made real-time data sharing on a large scale possible.
- Most communicable disease diagnosis and reporting occurs in private sector laboratories.

Public health laboratories provide these essential services: case finding in high-risk groups, outbreak detection, emergency response, environmental monitoring, and disease surveillance. Public and private laboratories have complementary (not competitive) roles in ensuring the health of their communities.

A national laboratory system would ensure the availability of consistent laboratory capacity for public health across the nation. Public health, hospital, and independent laboratories currently have a loose, inconsistent association; relationships between public and private partners are underdeveloped, and multiple barriers prevent information sharing. Federal initiatives have been categorical, rather than system building. Disease reporting is inadequate, and public health surveillance and response are compromised.

State and local public health laboratories can serve as a focal point for a national system, through their core functions (3): 1) disease prevention, control, and surveillance; 2) integrated data management; 3) reference and specialized testing; 4) environmental health and protection; 5) food safety; 6) laboratory improvement/regulation; 7) policy development; 8) emergency response; 9) public health related research; 10) training and education; and 11) partnerships and communication.

Hospital and independent laboratories have an important role to play as well, by participating in the system development process, submitting samples and isolates of public health importance, cosponsoring local, state, and national meetings, and helping develop standard methods and procedures for public health situations.

Professional organizations can contribute by helping develop and maintain state and regional databases of services, convening meetings of state and national laboratory network constituents, and working to produce a consensus process to determine which services will be available.

Federal agencies, especially CDC, must provide national leadership for system development in several ways: developing funding and reimbursement mechanisms; developing and promoting best practices guidelines; establishing an advisory body; developing and maintaining a Web-based information system that links CDC, public health, hospital, and independent laboratories; and focusing training needs on identified gaps.

Implementation of a national laboratory system for public health will hinge on state and regional initiatives and coordination. A consensus must be developed around the need for such a system, and the cooperation of public and private laboratories must be secured. Agreement must be reached about the roles of the respective participants, and federal agencies will need to provide active leadership. Examples of state and regional coordination activities might include (R.A. Martin, pers. comm., 2000):

- Establishing a collaborative network.
- Creating a menu of available services to support public health.
- Developing a state or regional database of constituent laboratories.
- Coordinating emergency response planning.
- Standardizing test methods for disorders and exposures of public health importance.
- Implementing active (vs. passive) surveillance systems
- Creating shared specimen delivery systems.
- Linking to other states, regions, and CDC.
- Building relationships with managed care organizations to ensure the flow of information and provision of specimens of public health importance.
- Addressing home and point-of-care testing issues that have public health implications.
- Establishing links to infectious disease physicians and infection control practitioners.
- Serving as an authoritative source on laboratory testing, as part of a national network.

### References

1. McDade J, Hughes J. The U.S. needs a national laboratory system. U.S. Medicine 1998;34:9.
2. Skeels M. Public health labs in a changing health care landscape. ASM News 1999;65:479-83.
3. Association of Public Health Laboratories. Core functions and capabilities of state public health laboratories. Washington: Association of Public Health Laboratories; 2000. p.1-13.

Address for correspondence: Michael R. Skeels, Oregon State Public Health Laboratory, P.O. Box 275, Portland, OR 97207, USA; fax: 503-229-5682; e-mail: Michael.r.skeels@state.or.us

